# Transmission Routes Analysis of SARS-CoV-2: A Systematic Review and Case Report

**DOI:** 10.3389/fcell.2020.00618

**Published:** 2020-07-10

**Authors:** Huanjie Li, Yangyang Wang, Mingyu Ji, Fengyan Pei, Qianqian Zhao, Yunying Zhou, Yatian Hong, Shuyi Han, Jun Wang, Qingxi Wang, Qiang Li, Yunshan Wang

**Affiliations:** ^1^School of Medicine, Cheeloo College of Medicine, Shandong University, Jinan, China; ^2^Medical Research and Laboratory Diagnostic Center, Jinan Central Hospital, Cheeloo College of Medicine, Shandong University, Jinan, China; ^3^Jinan Infectious Disease Hospital, Shandong University, Jinan, China; ^4^Basic Medical Research Center, Jinan Central Hospital, Cheeloo College of Medicine, Shandong University, Jinan, China

**Keywords:** SARS-CoV-2, transmission routes, eyes-oral transmission, asymptomatic patients, COVID-19

## Abstract

The global outbreak of SARS-CoV-2 spread rapidly throughout the world which transmitted among humans through various routes. Asymptomatic (carriers) and possible fecal-oral transmission, resulted into a large-scale spread. These issues pose great challenges to disease diagnosis and epidemic control. We obtained data on 29 cases of COVID-19 patients in Jinan, China, and reported the clinical data of asymptomatic patients confirmed with stool samples positive. Some patients with gastrointestinal infections are secondary to pulmonary infections, and during the patients' recovery period, the virus may still existin the patient's gastrointestinal tract over 7 days. We combined with epidemiological and clinical data of asymptomatic patients to analyze the possible routes of viral transmission and infection, including eyes-nose, hands-eyes, fecal-oral, and eyes-oral, et al., thus first presented the two-way transmission through eyes-oral. Through associating infection symptoms with the transmission routes of virus and the patient course of the disease, we expect to provide guidelines for clinical diagnosis and the basis for suppressing the spread of the virus and antiviral treatment.

## Introduction

In late December 2019, the global outbreak of a novel coronavirus (2019-nCoV) spread rapidly throughout the world resulting in more than 5,404,512 cases and 343,514 deaths till May 26th, 2020 (WHO, 2020). The numbers of confirmed cases are still rising, posing higher challenges for disease control and patient treatment. The new coronavirus was isolated from human airway epithelial cells and sequenced through the unbiased, high-throughput sequencing to identify microbial sequences (Zhu et al., 2020). Different from both MERS-CoV and SARS-CoV virus, 2019-nCoV was identified as the seventh member of the family of coronavirus that infects humans and has been formally named as the severe acute respiratory syndrome coronavirus 2 (SARS-CoV-2) by the International Committee on Taxonomy of Viruses. SARS-CoV-2 has strong transmission power, and it is easy to cause acute respiratory distress syndrome, septic shock, coagulation dysfunction, intestinal dysfunction, and other clinical symptoms post infection (Chen L. et al., 2020). At the onset of the illness, several patients were observed with extra-pulmonary manifestations, such as conjunctivitis, or even presented with asymptomatic infections (Rothe et al., 2020). But the potential routes of viral spread from patients or asymptomatic carriers to a healthy person has poorly understood (Zhang et al., 2020). Little was known about why and how the SARS-CoV-2 induced enteric symptoms, ocular diseases, or cardiac diseases. Scientific literature on SARS-CoV-2 infection is growing rapidly, and further research to determine the infectivity and viability of SARS-CoV-2 is essential to control its spread especially in asymptomatic carriers.

The entire genome of SARS-CoV-2 has been sequenced, and it encodes RNA polymerase, spike (S) glycoprotein, membrane (M) glycoprotein, envelope (E) glycoprotein, and nucleocapsid (N) proteins. SARS-CoV-2 possessed a typical genome structure of the coronavirus and belonged to the cluster of beta-coronaviruses (Lu R. et al., 2020). It has a close evolutionary association with SARS-CoV and MERS-CoV and is more than 82% nucleotide identity with SARS-CoV (Chan et al., 2020a). Although SARS-CoV-2 shares the same receptor ACE2 with SARS-CoV, there are some difference between the viruses in structure and function (Wan et al., 2020; Wu A. et al., 2020; Zheng and Song, 2020). The Cryo-EM structure of the SARS-CoV-2 S trimer in the profusion conformation was reported recently that ACE2 bound to SARS-CoV-2 S ectodomain with a higher affinity than binding to SARS-CoV. The high affinity may contribute to the apparent ease with which SARS-CoV-2 can spread from human to human (Wrapp et al., 2020). The estimated mean R0 for SARS-CoV-2 is around 3.28, with a median of 2.79 and IQR of 1.16, which is considerably higher than SARS-CoV (Liu Y. et al., 2020). Thus, ACE2 plays a vital role in the SARS-CoV-2 infection (Zhang et al., 2020). SARS-CoV-2 has evolved the stronger capability to infect and transmit among humans. To this day, there is no specific medicine to prevent or treat COVID-19. Studying on the viral transmission will provide significant information for understanding the pathogenesis of SARS-CoV-2 infection and preventing the massive spread of infectious diseases.

SARS-CoV-2 has been detected in multiple organs, for example, eyes, nasopharynx, saliva, alveolar lavage fluid, blood, intestine, feces after infection (Wang D. et al., 2020), which brings a great difficulty to reach the diagnosis. On February 1, 2020, respiratory samples of four patients were confirmed SARS-CoV-2 infections by real-time PCR in Jinan Central Hospital, Shandong province, China. Clinical characteristics and blood biochemical indexes of patients were recorded and examined at the hospital. On February 2, we collected samples of four patients' conjunctival secretions, feces, cell phone surfaces, hands surface, an inner surface of the mask, ground, door handles surface, the head surface of bed, and other samples in the isolation ward immediately. The SARS-CoV-2 nucleic acid for the above samples of patients was also detected immediately. Besides, we also collected the medical records of 29 confirmed patients during hospitalization from the isolation ward of Jinan Infectious Disease Hospital.

In this study, we systematically investigated the clinical and laboratory characteristics of confirmed 29 cases provided by Jinan infectious disease hospital, Shandong University, and the environmental samples collected from Jinan central hospital, Shandong University. Epidemiological and clinical characteristics of asymptomatic patients in Jinan are reported. Summarizing the published articles, including SARS-CoV and SARS-CoV-2, we combined with epidemiological and clinical data to analyze the possible routes of asymptomatic patients with virus infection in order to provide the basis for suppressing the spread of the virus, and antiviral treatment and advice for the protection of medical staff.

## Patients

Four patients were diagnosed with COVID-19 in Jinan central hospital, Shandong province. We collected throat swabs and blood samples and other environmental samples, including the four patients' conjunctival secretions, feces, cell phone surfaces, hands surfaces, an inner surface of the mask, ground, door handles surface, the head surface of bed, et al. in the isolation ward before they were transferred to Jinan infectious disease hospital. In 2 weeks, there were 29 cases of COVID-19 patients transferred to Jinan infectious disease hospital for hospitalization, including the above-mentioned four patients. All the cases were confirmed by RT-PCR and were analyzed for epidemiological, clinical, radiological features, and laboratory data.

This study was approved by the Ethics Commission of Jinan central hospital affiliated to Shandong University and Jinan infectious disease hospital affiliated to Shandong University. The patients waived the right to informed consent.

## Methods

We recorded and analyzed the contact history, hematological, biochemical, radiological, and microbiological investigation results. The respiratory tract, blood, and stool samples of 29 patients were collected. RNA of biological and environmental samples were extracted using Liferiver (Shanghai Liferiver Biotechnology Co., Ltd.) Nucleic Acid Extraction Kit, which had been registered for detecting ORF1ab gene, E gene, and N gene of SARS-CoV-2. Samples were tested by RT-PCR following the steps of the kit in a tertiary protection laboratory.

Respiratory samples of the patients were also tested for influenza A and B viruses and respiratory syncytial virus using the Xpert Xpress Flu/RSV assay (GeneXpert System) according to the instructions.

### Statistical Analysis

By searching Pubmed Web, we analyzed and compared the reports of SARS-CoV, MERS-CoV, and SARS-CoV-2 in different countries in the early outbreak stage. Local case analysis in this paper adopts the method of descriptive statistics, tables, and graphs.

## Results

Compared with Wuhan, the 29 cases of COVID-19 in Jinan exhibited mild or moderate symptoms, which are mainly imported cases from Wuhan's contact history (Tables 1, 2). Of the 29 cases, 44.8% had a history of contact with Wuhan person, while others were clustering disease. The average age of the patients was 38.2 years old (SD = 13), including male (11[37.9%]) and (18[62.1%]) female. SARS-CoV-2 nucleic acid was detected in all patients by real-time RT-PCR. But two of them were asymptomatic, and they were compulsively detected nucleic acid because of close contact with confirmed cases. Asymptomatic patients were also treated in hospital's isolation wards until their detection turned negative, with one patient of them showing stool positive before being discharged.

**Table 1:** The epidemiological, clinical and radiological features and laboratory data of SARS-CoV-2 confirmed patients in Jinan.

**Number**	**Age**	**Sex**	**Admission date**	**Days of onset**	**Symptom**	**CT**	**Epidemiology**	**Stool sample of SARS** **-CoV-2**	**FluA,B and RSV**	**Blood sample of SARS** **-CoV-2**
1	29	Female	23/Jan/2020	16	Fever 37.8°C	Bilateral pneumonia	Wuhan contact history	Negative	Negative	Negative
2	26	Male	26/Jan/2020	12	Fever 37.8°C, cough, pharyngalgia	Bilateral pneumonia	Wuhan contact history	Negative	Negative	Negative
3	32	Male	26/Jan/2020	14	Fever 38°C, pharyngalgia	Bilateral pneumonia	Wuhan contact history	Negative	Negative	Negative
4	36	Female	27/Jan/2020	10	Fever 38.1°C	Bilateral pneumonia	Wuhan contact history	Negative	Negative	Negative
5	27	Female	26/Jan/2020	6	Symptomless	Symptomless	Clustering disease in family	**Positive**	Negative	Negative
6	60	Female	26/Jan/2020	6	Symptomless	Pneumonia (Left lung)	Clustering disease in family	Negative	Negative	Negative
7	39	Female	27/Jan/2020	7	Fever 37.6°C	Bronchitis	Clustering disease in family	**Positive**	Negative	Negative
8	56	Female	27/Jan/2020	5	Symptomless	Pneumonia (Left lung)	Clustering disease in family	Negative	Negative	Negative
9	30	Male	28/Jan/2020	4	Symptomless	Symptomless	Clustering disease in family	Negative	Negative	Negative
10	30	Male	29/Jan/2020	6	Fever 37.2°C	Bilateral pneumonia	Wuhan contact history	Negative	Negative	Negative
11	35	Male	29/Jan/2020	7	Fever 38°C, muscle ache	Bilateral pneumonia	Wuhan contact history	Negative	Negative	Negative
12	33	Female	30/Jan/2020	3	Fever 38.3°C, pharyngalgia, rhinobyon	Pneumonia (Right lung)	Wuhan contact history	Negative	Negative	Negative
13	8	Female	30/Jan/2020	3	Fever 38°C, cough	Pneumonia (Right lung)	Clustering disease in family	Negative	Negative	Negative
14	58	Female	31/Jan/2020	8	Muscle ache, loss of appetite, general fatigue	Pneumonia (Right lung)	Wuhan contact history	**Positive**	Negative	Negative
15	35	Male	31/Jan/2020	4	Fever 37.7°C	Bilateral pneumonia	Wuhan contact history	Negative	Negative	Negative
16	35	Male	01/Feb/2020	1	Symptomless	Bronchitis	Clustering disease in family	Negative	Negative	Negative
17	33	Female	02/Feb/2020	2	Cough	Pneumonia (Left lung)	Wuhan contact history	Negative	Negative	Negative
18	55	Female	02/Feb/2020	1	Cough	Bronchitis	Clustering disease	Negative	Negative	Negative
19	40	Male	02/Feb/2020	N/A	Fever38°C, muscle ache, general fatigue	Bilateral pneumonia	Clustering disease	Negative	Negative	Negative
20	40	Male	03/Feb/2020	N/A	Fever 37.5°C, chest stuffiness	Bilateral pneumonia	Clustering disease	Negative	Negative	Negative
21	59	Female	03/Feb/2020	N/A	Symptomless	Pneumonia (Right lung)	Clustering disease	**Positive**	Negative	Negative
22	37	Male	03/Feb/2020	N/A	Fever 38°C	Pneumonia (Right lung)	Wuhan contact history	Negative	Negative	Negative
23	39	Female	04/Feb/2020	13	Fever 37.3°C, cough, chest pains	Symptomless	Wuhan contact history	Negative	Negative	Negative
24	39	Female	04/Feb/2020	10	N/A	Bilateral pneumonia	Clustering disease	Negative	Negative	Negative
25	22	Female	04/Feb/2020	N/A	Fever 38.5°C, cough, white phlegm	Bilateral pneumonia	Clustering disease	Negative	Negative	Negative
26	49	Female	04/Feb/2020	5	Fever 37.6°C, cough, sputum	Bilateral pneumonia	Clustering disease	Negative	Negative	Negative
27	71	Male	05/Feb/2020	3	Fever 38°C	Bilateral pneumonia	Clustering disease	Negative	Negative	Negative
28	50	Female	05/Feb/2020	0	Symptomless	Pneumonia (Right lung)	Clustering disease	Negative	Negative	Negative
29	28	Female	05/Feb/2020	N/A	Fever 37.3°C, muscle ache	Symptomless	Wuhan contact history	Negative	Negative	Negative

**Table 2:** Percentage of clinical symptoms in Jinan SARS-CoV-2 confirmed patients in Jinan.

	**Total**	**Male**	**Fever**	**Cough or pharyngalgia**	**Muscle ache**	**Pneumo-nia**	**Symptomless**	**Wuhan contact history**	**Stool sample of SARS-CoV-2 positive**
Total	29	11	18	9	4	25	4	13	4
Percent	100.0%	37.9%	62.1%	31.0%	13.8%	86.2%	13.8%	44.8%	13.8%

**Figure 1:**
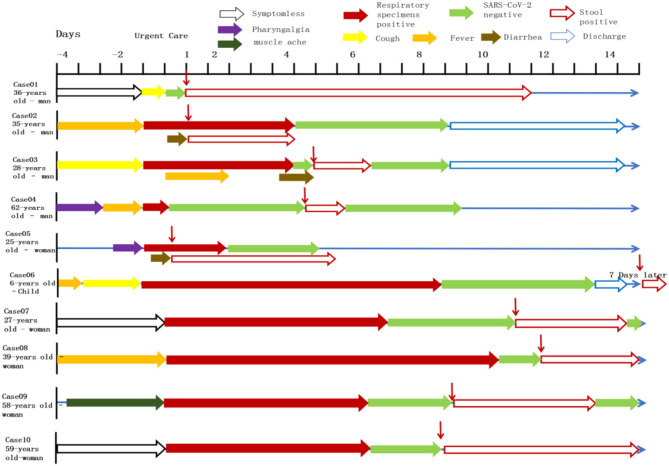
Symptoms according to day of illness and hospitalization among the cases confirmed SARS-CoV-2. Representative cases were combined the timeline of positive throat and anal swabs. Case07-10 were treated in Jinan infectious disease hospital and the remaining cases were reported in other hospitals. The patients are some particular cases of being discharged. After the throat swab turned to negative, the stool samples of patients became positive again appeared 5–7 days after discharged, or even longer.

**Figure 2:**
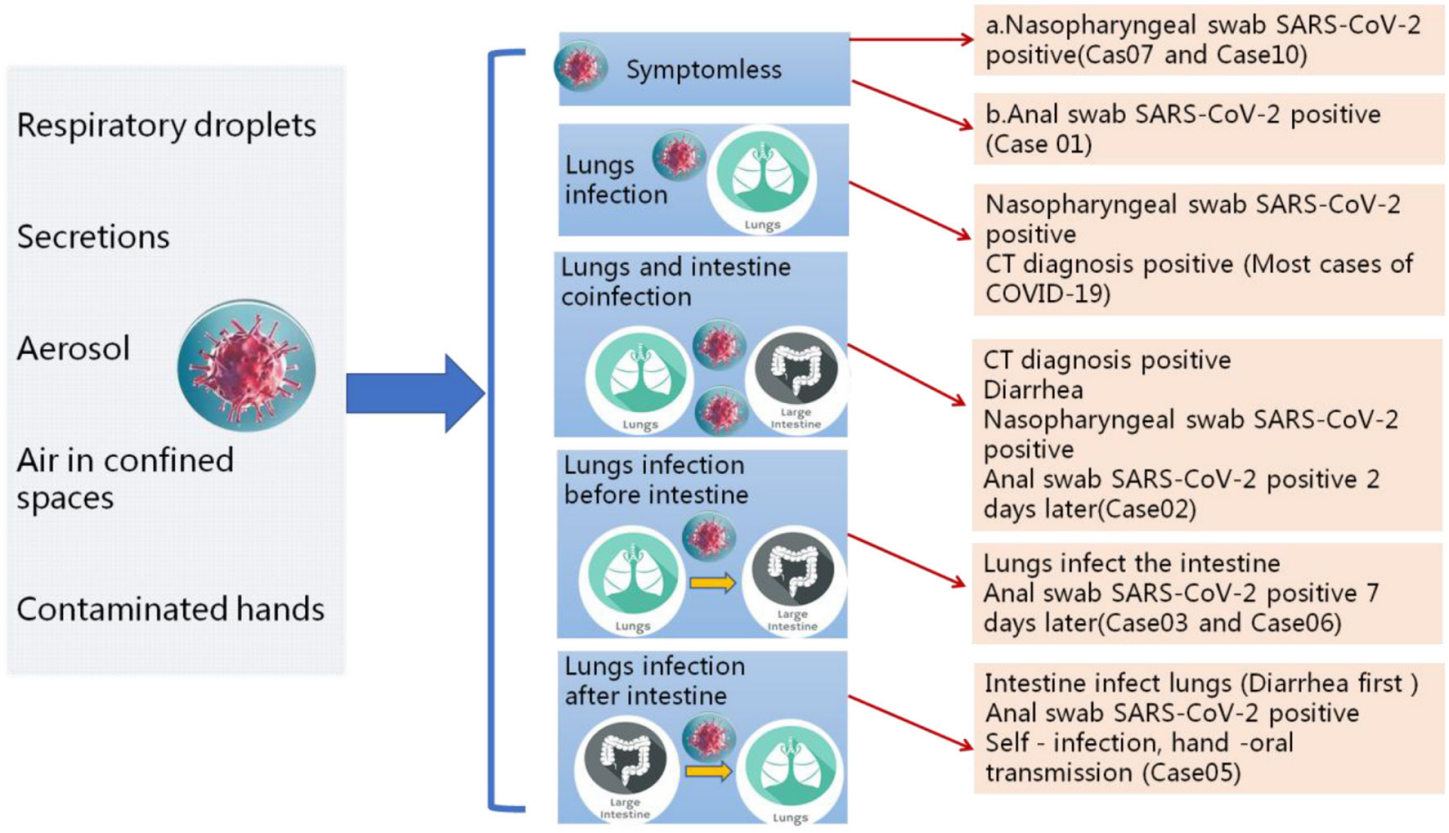
Different clinical symptoms reflect different transmission pathways of SARS-CoV-2. Patients with different clinical symptoms may be closely related to the route of the virus during transmission. The infection sites of virus causing different clinical symptoms and directly affects the course of disease.

All patients were tested for the nucleic acid of Influenza A and B viruses (FluA, B) and respiratory syncytial virus (RSV). We did not find the co-infection of SARS-CoV-2 and Flu A, B, or RSV viruses in the patients because of the low sample size. Patients had clinical manifestations of fever (18 [62.1%] patients), cough and pharyngalgia (9 [31%] patients), pneumonia (25 [86.2%] patients), muscle aches (4 [13.8%] patients), fecal nucleic acid positive (4 [13.8%] patients). According to imaging examination, 25 [86.2%] patients showed pneumonia (Tables 1, 2). We sorted out 10 representative cases and combed the timeline of the positive throat and stool samples (Figure 1). Four cases are enrolled in Jinan, and the rest of the cases are reported by other agencies. In addition to patients whose initial symptoms of infection are diarrhea, there are some particular cases. After the throat swab turned to negative, their stool samples became positive again, and most of the clinical manifestations appeared 5–7 days after the pulmonary symptoms. Some patients with gastrointestinal infections were secondary to pulmonary infections, and during the patients' recovery period, the virus may still be released from the patient's gastrointestinal tract for 7 days, or even longer. We inferred virus being excreted through the intestine may be more beneficial to the recovery of patients. The replication and duration of SARS-CoV-2 detoxification is directly related to the prognosis of patients. Patients with different clinical symptoms may be closely related to the route of the virus during transmission. The infection sites of virus causing different clinical symptoms and directly affects the course of disease. We speculated several different clinical symptoms may reflect various transmission pathways of SARS-CoV-2 shown in Figure 2. The symptomless SARS-CoV-2 carriers were divided into two groups, i.e., the throat swab positive or the stool sample positive, which reflected the different target sites of a susceptible patient. Study on the first clinical symptoms and the progression of the disease, lungs-intestine transmission route showed that there are three kinds of routes: from the lungs to intestine, the self-infection from the intestine to lungs, co-infection of lungs, and intestine.

Combing the time points of patients' hospitalization may be helpful to provide a guideline for COVID-19 diagnosis and treatment. Whether the excretion of feces *in vitro* during the recovery period is infectious remains to be further studied. The criteria of patients discharged should be reassessed, and the nucleic acid testing of anal swabs or stool samples should be added.

Environmental samples detection of four patients with early diagnosis, including eye and conjunctival secretions, telephone surfaces, hands surface, masks, the surface of door handles and bed at home, and other samples in the isolation ward, were all negative except their masks. The virus nucleic acid testing of the blood and stool samples were all negative on admission. But the nucleic acid test of their stool samples became positive after the patients were hospitalized for 8–10 days, while nucleic acid test of throat swab turned to negative.

SARS-CoV-2 is mainly transmitted through the respiratory tract in the early stage and can be survival longer on the mask. The epidemiological survey showed the four patients had no recurrence of human transmission before isolated by the hospital.

Cases in Jinan are mainly mild symptoms, which indicated that timely diagnosis, early detection, early isolation are very effective approaches before the virus becomes highly contagious. There are no new cases reported for the 26 successive days in Jinan. The fundamental measure for controlling infectious diseases is to cut the source of infection. It is suggested that study on the transmission routes of the virus is a feasible pathway in controlling the disease effectively and we summarize the transmission routes of SARS-CoV-2 systematically.

### The Transmission of Respiratory Droplets and Mucous Membranes Infections Are the Main Route of SARS-CoV-2, but Not the Only One

Just like SARS-CoV, SARS-CoV-2 is a typical respiratory virus causing highly contagious potentially lethal disease. The mucous membranes infection is still the main transmission. Many clinical cases developed influenza-like symptoms, with a 2–4 days history of cough and subjective fever (Wang D. et al., 2020; Wu F. et al., 2020; Zhu et al., 2020). Coronavirus gains entry into host cells through recognizing and binding to the host receptor ACE2 distributed from the conjunctiva (Wan et al., 2020). Once exposed directly or indirectly to the virus (infectious droplets, body fluids, virus-carrying hands), the mucosal cells in the conjunctiva, mouth, nasal cavity, or throat were susceptibility infected by the virus for replicating (Gao et al., 2016; Zhou P. et al., 2020). A larger amount of virus was assembled and released into the human lungs through the respiratory tract, resulting in various types of fever, cough, or ground-glass opacity of lung on CT examination results and even respiratory failure.

**Figure 3:**
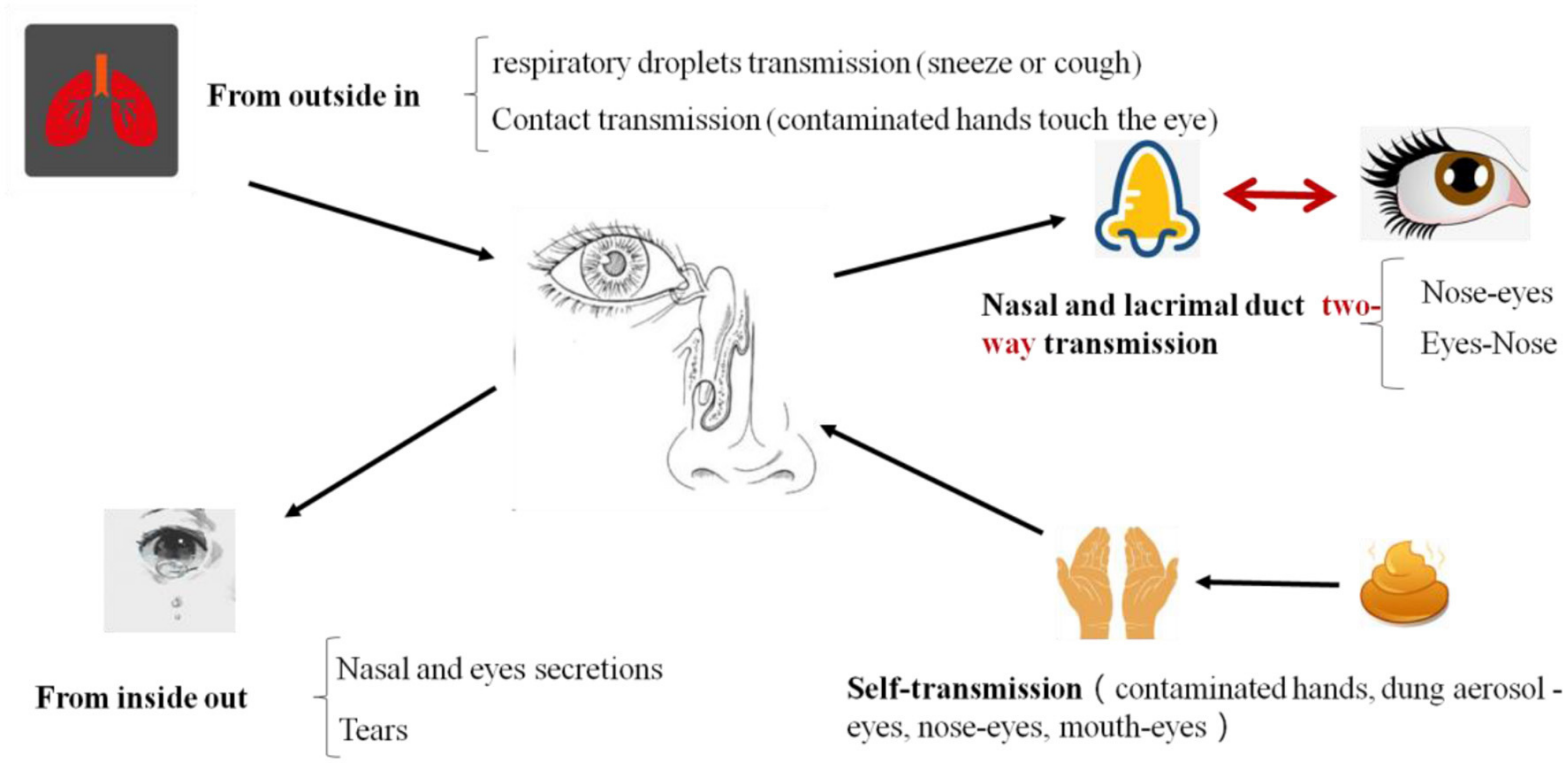
The routs of SARS-CoV-2 infection and transmission in eyes. Eyes are important portals of entry for virus. After SARS-CoV-2 infected and replicated in eyes, it will be transmitted through two ways: one is outward transmission, eye secretions, or tears with virus contaminate the hands, and then there is a risk of transmission of the virus through hands. Another route is inward transmission. If the virus infected person though eyes, conjunctival secretions, and tears can flow into the mouth through the nasopharyngeal tube then reach the lungs or gastrointestinal tract and more infections occur.

**Figure 4:**
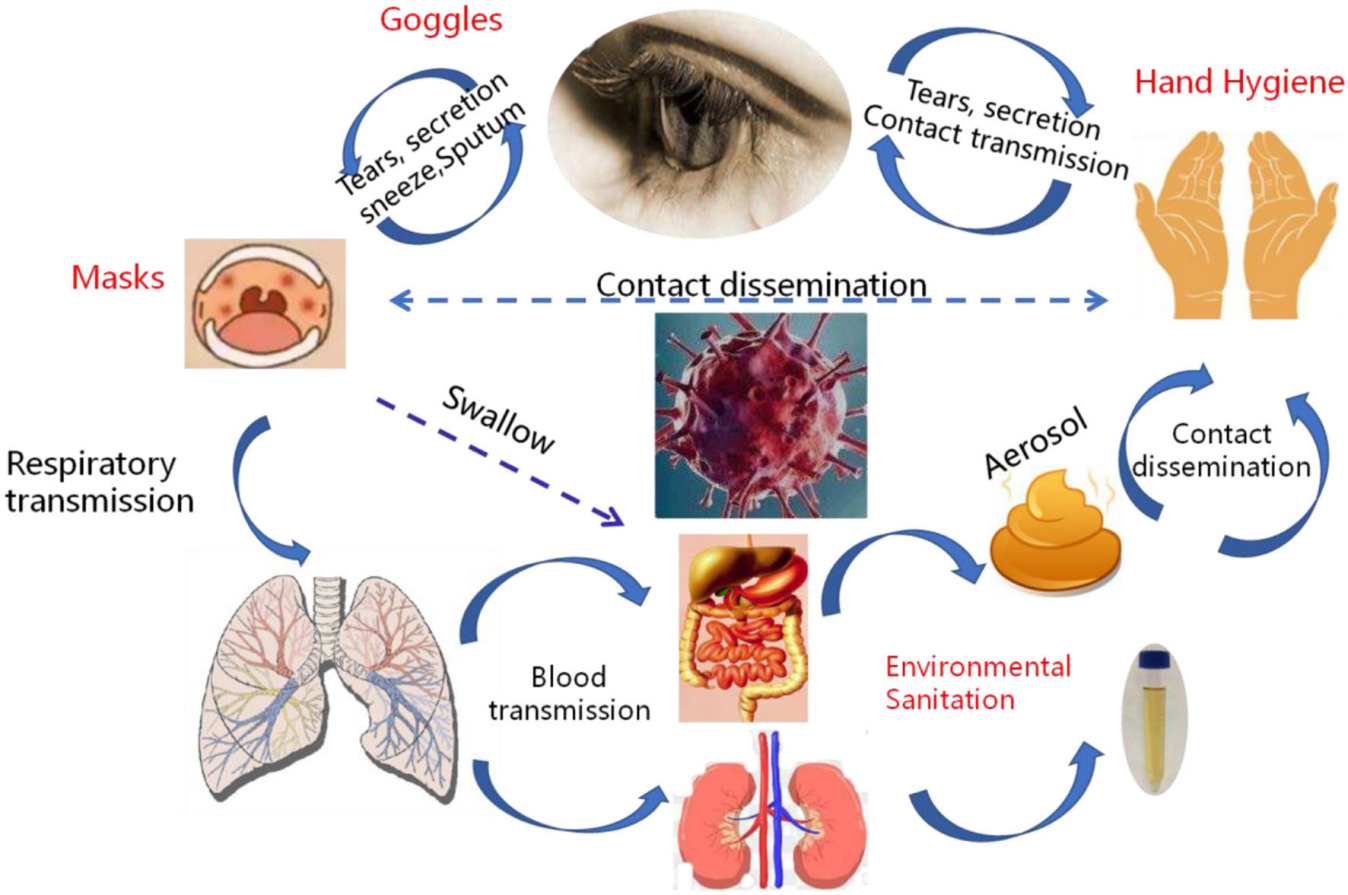
The transmission pathways of the SARS-CoV-2 *in vitro* and *in vivo*. Although direct droplet transmission is the main route route of transmission, fecal excretion, environmental contamination, and fomites might contribute to viral transmission. The prevention and control principles of infectious diseases should include the control of spatter and droplet transmission, contact transmission, fecal-eye transmission, nasal-eye transmission, oral-eye transmission, and the transmission of ocular secretions and tears.

The SARS-CoV-2 mainly spread from person to another through small respiratory droplets from the nose or mouth when a person confirmed COVID-19 coughs or exhales (Figures 3, 4). These droplets land on objects and surfaces around the person. Other people may catch the virus by breathing in droplets or touching these objects or surfaces, then touching their eyes, nose, or mouth. The risk of catching SARS-CoV-2 from someone with no symptoms is very low. However, many people with SARS-CoV-2 experience only mild symptoms, particularly true in the early stages of the disease. Some cases reported that conjunctivitis was the first symptom and they were infected while had a history of close contact with a patient with COVID-19 (Chen L. et al., 2020; Lu C. W. et al., 2020). It speculates that there may be a risk of tears and conjunctival transmission. Growing evidence shows that the virus attacks multiple organs in the body.

### Eye Infection and the Ocular Route: A Specific Transmission That Should Not Be Ignored, Might Be Another Route to Lung Infection

During the outbreak of SARS-CoV-2, some patients developed symptoms of conjunctivitis. Some patients even suffered the ocular diseases in clinical diagnosis before fever and cough (Chen L. et al., 2020). There have been case reports in which many ophthalmologists were found to be infected through routine diagnosis and treatment with only his eyes unprotected (Chan et al., 2020a; Xia et al., 2020). Therefore, if conjunctivitis as the initial symptom of confirmed COVID-19 patients was neglected and contacted without comprehensive measures, the infectious tears and body fluids containing the virus could infect other persons (Belser et al., 2013; Lu C. W. et al., 2020). Those results suggested that the eyes route of transmission existed (Chan et al., 2020b; Huang et al., 2020). Deng et al. (2020) demonstrated that macaques can be infected with SARS-CoV-2 via the conjunctival route.

We proposed that the SARS-CoV-2 transmitted and infected through eyes including two routes (Figure 3). One is direct contact and the other is indirect contact transmission. The direct route is that droplets with virus enter through the eyes. For example, in a fever clinic, a relatively closed environment, there is a high risk of eye infections. When medical personnel performs close-up operations, virus droplets (aerosols pollution) will spray out which may splash into the eyes and cause infections, so medical staff are strongly recommended wearing goggles or a mask. A virus presented in these body fluids may affect our precautionary practices and sites of sampling for diagnostic tests. Recently, Jiang et al. found that the virus was present both on surfaces and in the air (Jiang et al., 2020). The two positive areas were the surfaces of the nurse station in the isolation area with suspected patients and the air of the isolation ward with an intensive care patient. So high contraction of nucleic acid may exist in the aerosol and influence operator, even the test result. Another route is through indirect contact infection, that is, accidentally touching the virus droplets with your hands and rubbing your eyes or noses may cause conjunctival infection.

Therefore, once SARS-CoV-2 infected and replicated in eyes, it will be transmitted through two ways (Figure 3), one is outward transmission, eye secretions, or tears with virus contaminating the hands, and then there is a risk of viral transmission through hands. Another route is an inward transmission. If the virus infects person through eyes, conjunctival secretions, and tears can flow into the mouth through the nasopharyngeal tube which will ultimately reach the lungs or gastrointestinal tract and more infections may occur.

Routes of transmission of SARS-CoV-2 other than respiratory droplets and stool are still enigmatic. The proportion of patients confirmed SARS-CoV-2 with conjunctivitis is much smaller than respiratory symptoms, which reflect that the eyes are not the most important organ for propagating the virus. For instance, the eyes cannot generate infectious aerosol unless an eye ezamination has performed. But we still insist that the eyes are important portals of entry for virus.

Moreover, increasing reports each day suggesting that SARS-CoV-2 cases began with eye redness and tingling as the leading symptoms, and the literature suggests that viruses can infect the human body through conjunctiva (Lu C. W. et al., 2020; Wang W. et al., 2020). These results showed that a few new cases of COVID-19 began with conjunctivitis as the first symptom, and the SARS-CoV-2 containing in the eye surface may enter into the nasal cavity and throat through drain tears.

SARS-CoV-2 can be transmitted through the nose-eye, possibly through the way of increased oral pressure caused by coughing or sneezing, and reverse transmission of the virus through the nasolacrimal duct to the dacryocyst and then infect the conjunctival cornea (Sun et al., 2020; Zhou Y. et al., 2020). Thus, this route is a two-way transmission route (Figure 4). The lacrimal route, via drainage of tear fluid including virus from punctum in the upper and lower eyelid through canaliculi to the lacrimal sac, and further through the nasolacrimal duct to the nasal cavity, would be another pathway available for SARS-CoV-2 infection. During replication in the ocular tract there will be a continuous influx of virions to the nasal cavity, and respiratory infection may be established. The possibility of subclinical and/or prolonged virus replication in the eye, followed by continuous transfer to the respiratory tract cannot be excluded.

### Fecal-Oral Transmission Route of SARS-CoV-2 Was Closely Related to Discharge Standard and Should Not Be Neglected

Generally, many respiratory pathogens, such as influenza, SARS-CoV and MERS-CoV, cause enteric symptoms, so is SARS-CoV-2 (Holshue et al., 2020). Diarrhea was observed in a considerable number of patients. In early reports from Wuhan, 2–10% of patients with COVID-19 had gastrointestinal symptoms such as diarrhea or vomiting (Chen N. et al., 2020; Wang D. et al., 2020). Abdominal pain was reported more frequently in patients admitted to the intensive care unit than in individuals who did not require intensive care unit care, and 10% of patients presented with diarrhea and nausea 1–2 days before the development of fever and respiratory symptoms (Yeo et al., 2020).

The study found that the detection of SARS-CoV-2 nucleic acid positive in a few feces of patients with confirmed COVID-19 cases indicated the presence of a live virus. Nanshan Zhong and Lanjuan Li teams have isolated SARS-CoV-2 from the fecal swab specimens of the pneumonia patient with COVID-19 separately. These findings demonstrated the presence of live viruses in the feces of patients. The recent occurrence of two COVID-19 patients in the same building in HongKong also provide the evidence of fecal transmission. Indeed, the SARS-CoV-2 receptor ACE2 is highly expressed on differentiated enterocytes. SARS-CoV-2 can infect enterocyte lineage cells in a human intestinal organoid model (Lamers et al., 2020).

Fecal transmission mode accounts for a small proportion of respiratory virus transmission. Most of the virus transmitted through the feces are enteroviruses, and respiratory viruses are mainly transmitted through droplets and contact. However, more than 15% of cases showed that the anal test of several patients had become positive at the later stage, while the chest radiographic evidence and viral clearance in respiratory samples from the upper respiratory tract showed significant improvement, so fecal formation route cannot be ignored. Pan et al. (2020) reported that the viral loads of stool samples were less than those of respiratory samples, so whether the excretion of feces *in vitro* during the recovery period is infectious remains to be further studied.

Considering the evidence of fecal excretion for SARS-CoV-2, the virus can also be transmitted via the fecal-oral transmission route or re-transmitted through the formation of aerosols in virus-containing feces. The transmission route of the tract may not be a single transmission. It may be a medium channel for other routes (Figure 4). Therefore, the standard procedure of stool sample collection and examination in patients with SARS-CoV-2 is important to protect medical staff and reduce the risk of infection.

### The Blood-Borne Spread of SARS-CoV-2 May Be Caused by Cytokine Storms (CSS)

Cytokines (CK) are key factors regulating the immune response caused by many infectious pathogens that can significantly damage host organs and tissues. In the early stages of SARS-CoV infection, cytokine levels in the blood, such as Il-6, Il-8, and TNF-α, are rapidly elevated, the elevation of which is associated with the progression of lung invasion and injury (Wong et al., 2004). MERS-CoV infection was also reported inducing increased concentrations of proinflammatory cytokines (IFNγ, TNFα, IL15, and IL17) (Mahallawi et al., 2018).

Huang et al. reported 41 patients with laboratory confirmed SARS-CoV-2 infection and those high concentrations of cytokines recorded in plasma of critically ill patients (Huang et al., 2020). The concentrations of various cytokines (Il-6) in the blood of severe patients with SARS-CoV-2 infection were significantly higher than those of non-serious patients. The concentration of cytokines can indicate the severity of the disease. Liu et al. analyzed the blood immunological indexes of 33 patients with new coronary pneumonia and found that after SARS-CoV-2 infection, the pathogenic T cells were quickly activated to produce granulocyte-macrophage colony stimulating factor (GM-CSF) and IL-6 (Liu J. et al., 2020). GM-CSF further activates CD14, CD16 inflammatory monocytes, producing more IL-6 and other cytokines, resulting in a cytokine storm that leads to severe immune damage to the lungs and other organs. More and more cases reported that the brain, kidney, and heart impairment induced by the virus infection, may contribute to multi-organ failure and death eventually (Li et al., 2020; Wu C. et al., 2020).

**Figure 5:**
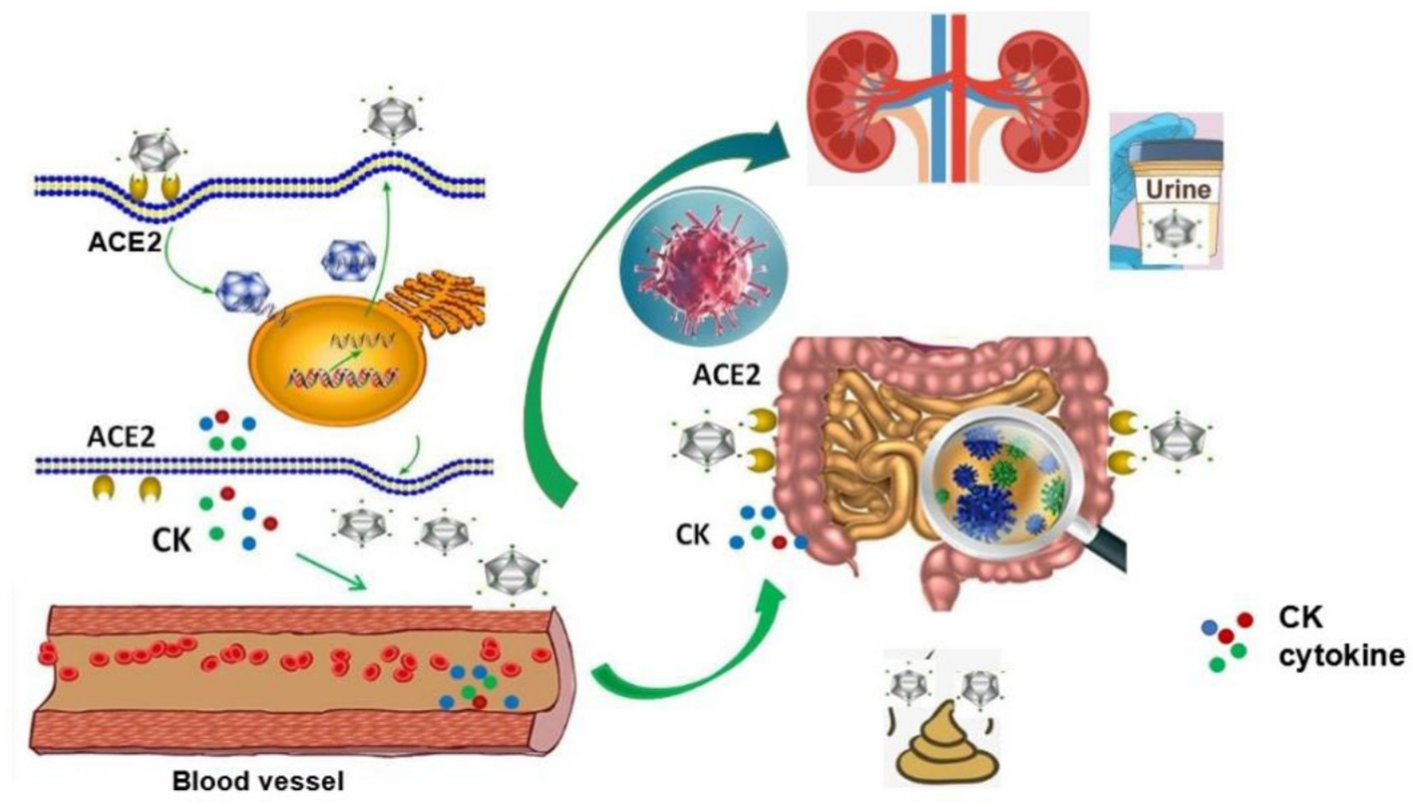
Possible mechanisms of blood transmission after viral infection. After SARS-CoV-2 enters into the lung from mouth and throat and infects cells, the virus replicates in the cell and releases more new viruses. Massive accumulation of SARS-CoV-2 leads to a surge of immune cells and pro-inflammatory cytokines, which resulting in a rapid increase in CK levels in the blood, or releasing more virus particles into the blood circulation. The virus and cytokines positively induce high expression of ACE2 in the intestinal epithelium and other organs, which accelerates overexpression of ACE2 and viral binding, causing the systemic infections with the virus.

Those cases with SARS-CoV-2 indicate that there is a dissemination way (lymphatic, hematogenous, direct invasion of adjacent tissues, and pathogenic implantation) in blood vessels of viral infection, which usually occurs in critical patients. This potential way for the viral spread is that SARS-CoV-2 enters into the lung from mouth and throat and then infects cells. The virus replicates in the cell and releases more new viruses. Massive accumulation of SARS-CoV-2 leads to a surge of immune cells and more and more pro-inflammatory cytokines, resulting in a rapid increase in CK levels in the blood, or releasing more virus particles into the blood circulation. The virus and cytokines positively induce high expression of ACE2 in the intestinal epithelium and other organs which accelerated over-expression of ACE2 and viral binding, causing systemic infections with the virus (Figure 5). The model might explain why the SARS-CoV-2 nucleic acid testing in the stool of some patients turns to positive occurs in the later days of treatment.

## Discussion

The ongoing outbreak of SARS-CoV-2 causes an epidemic of acute respiratory syndrome in humans in Wuhan, China. It has rapidly spread to national regions and other countries, thus, pose a serious threat to public health. Our research reported the characteristics of SARS-CoV-2 epidemiology in Jinan. Compared with Wuhan, 29 cases of COVID-19 in Jinan exhibited mild or moderate symptoms, which are mainly imported cases from Wuhan's contact history. The infection and transmission capacity of the virus is greatly weakened, which may be due to the fact that the cases in Jinan are mostly second or third generation communicators.

**Figure 6:**
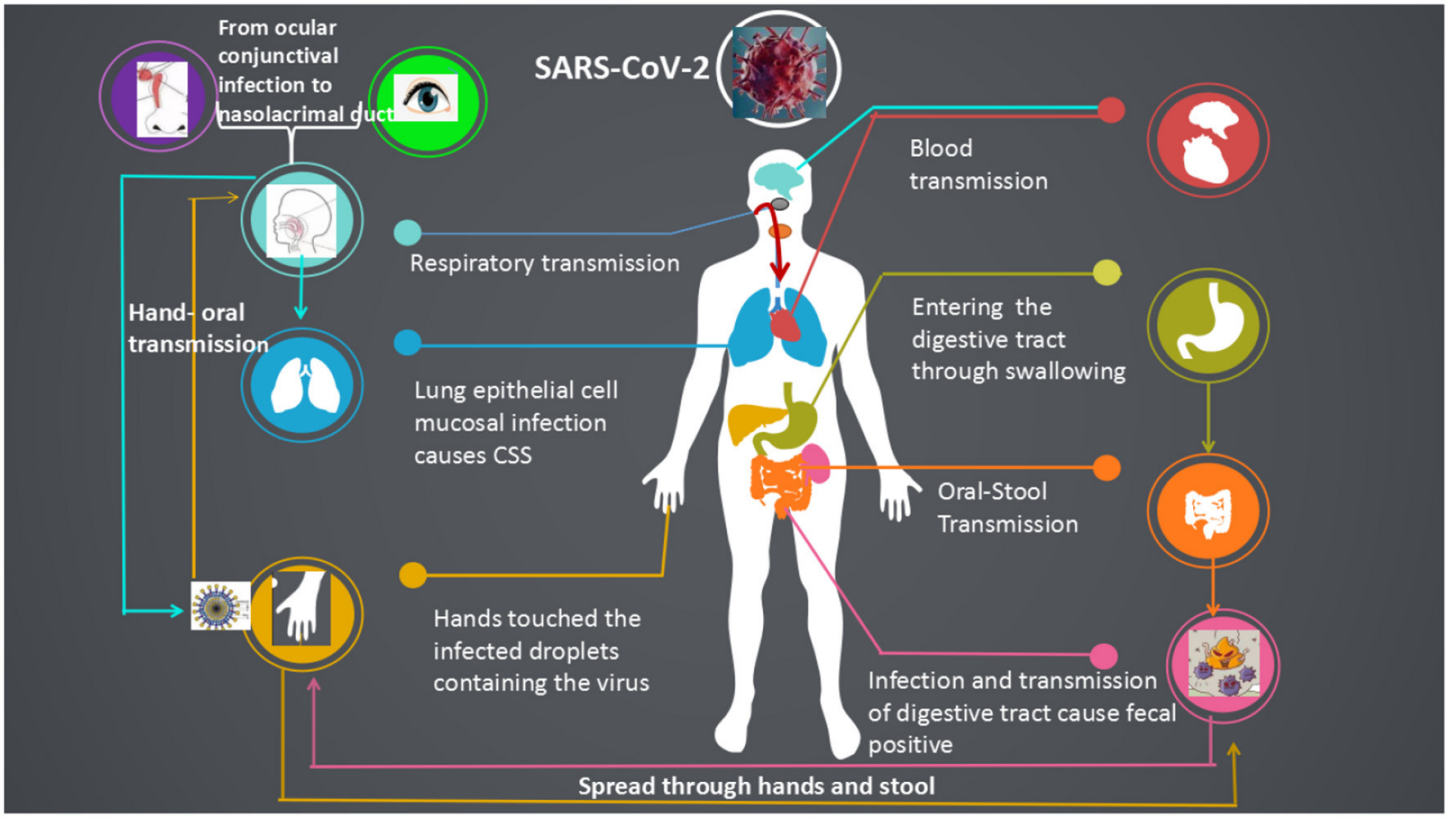
Systemic transmission pathways and susceptible organs of the SARS-CoV-2. SARS-CoV-2 was transmitted in a more diverse way among human, respiratory transmission, fecal-oral, hand-oral, eye-nose-oral, stool-hands- oral transmission and blood transmission.

The incubation period of SARS-CoV-2 is 1–14 days, which is infectious and the incubation period is equally contagious. The main routes of viral transmission are respiratory tract, including the spatter (e.g., blood spatter, spatter during intubation, etc.) and droplet transmission (sneezing or cough), contact transmission (e.g., hand wiping eyes), fecal-eye transmission, nasal-eye transmission, mouth-eye transmission (through contaminated hands or objects) and the transmission of eye secretions and tears (Figure 6). If the virus first contacts the conjunctiva of the patients' eyes, or the hand touches the virus and rubs the eye, the virus will invade the patient's eye conjunctiva, infect and reproduce, causing eye swelling which can even lead to conjunctivitis. The replicated virus may pass through the tear fluid to patient's nasolacrimal duct and enter the respiratory infection pathway when coughing or enter the digestive tract infection pathway when swallowing food. It insisted that the eyes are important entrance and replication sites of SARS-CoV-2.

Although direct droplet transmission is the main route of transmission, fecal excretion, environmental contamination, and fomites might contribute to the viral transmission. Considering the evidence of fecal excretion, SARS-CoV-2 can also be transmitted via the fecal-oral transmission route. The virus can stay active in the digestive tract for more than 7 days, even longer time than in the lungs. After the throat swab turns negative, SARS-CoV-2 can also be detected in the feces of patients. Investigating on the infection and transmission of the virus, we reported the asymptomatic person with SARS-CoV-2 positive. It is not clear whether the virus discharged from the feces is infectious or not, the body excrete the virus through the feces is a beneficial way for human self-regulation. The replication and duration of virus detoxification may be directly related to the prognosis of patients. Besides, when pulmonary infection secondary to intestinal infection, patients should also avoid the occurrence of self-infection. In high prevalence season of influenza, it may be necessary to detect the influenza virus if diarrhea appeared, to avoid the occurrence of fecal-oral transmission and lung infection. Moreover, asymptomatic carriers could acquire and transmit the SARS-CoV-2, which makes the prevention of COVID-19 infection great challenge in the world.

## Data Availability Statement

All datasets generated for this study are included in the article/supplementary material.

## Ethics Statement

This study was approved by the Medical Ethics Committee of Jinan Central Hospital Affiliated to Shandong University. The committee waived the need for specific consent.

## Author Contributions

HL and YaW conceived and wrote the manuscript. HL and YaW drew the pictures. YH and MJ collected patient's clinical information. QL provided the cases. QZ, YZ, FP, and SH did the nucleic acid testing of SARS-CoV-2. YuW and QW managed the project. All other authors contributed to the analysis, reviewed results, and reviewed the manuscript.

## Conflict of Interest

The authors declare that the research was conducted in the absence of any commercial or financial relationships that could be construed as a potential conflict of interest.

## References

[B1] Belser J. A. Rota P. A. Tumpey T. M. (2013). Ocular tropism of respiratory viruses. Microbiol. Mol. Biol. Rev. 77, 144–156. 10.1128/MMBR.00058-12 23471620 PMC3591987

[B2] Chan J. F. Kok K. H. Zhu Z. Chu H. To K. K. Yuan S.. (2020a). Genomic characterization of the 2019 novel human-pathogenic coronavirus isolated from a patient with atypical pneumonia after visiting Wuhan. Emerg. Microbes Infect. 9, 221–236. 10.1080/22221751.2020.1719902 31987001 PMC7067204

[B3] Chan J. F. Yuan S. Kok K. H. To K. K. Chu H. Yang J.. (2020b). A familial cluster of pneumonia associated with the 2019 novel coronavirus indicating person-to-person transmission: a study of a family cluster. Lancet 395, 514–523. 10.1016/S0140-6736(20)30154-9 31986261 PMC7159286

[B4] Chen L. Liu M. Zhang Z. Qiao K. Huang T. Chen M.. (2020). Ocular manifestations of a hospitalized patient with confirmed 2019 novel coronavirus disease. Br. J. Ophthalmol. 104, 748–751. 10.1136/bjophthalmol-2020-316304 32265202 PMC7211077

[B5] Chen N. Zhou M. Dong X. Qu J. Gong F. Han Y.. (2020). Epidemiological and clinical characteristics of 99 cases of 2019 novel coronavirus pneumonia in Wuhan, China: a descriptive study. Lancet 395, 507–513. 10.1016/S0140-6736(20)30211-7 32007143 PMC7135076

[B6] Deng W. Bao L. Gao H. Xiang Z. Qu Y. Song Z. (2020). Ocular conjunctival inoculation of SARS-CoV-2 can cause mild COVID-19 in Rhesus macaques. bioRxiv [Preprint]. 10.1101/2020.03.13.990036 PMC7467924 32879306

[B7] Gao H. Yao H. Yang S. Li L. (2016). From SARS to MERS: evidence and speculation. Front. Med. 10, 377–382. 10.1007/s11684-016-0466-7 27726088 PMC7088694

[B8] Holshue M. L. DeBolt C. Lindquist S. Lofy K. H. Wiesman J. Bruce H. (2020). First case of 2019 novel coronavirus in the United States. N. Engl. J. Med. 382, 929–936. 10.1056/NEJMoa2001191 32004427 PMC7092802

[B9] Huang C. Wang Y. Li X. Ren L. Zhao J. Hu Y.. (2020). Clinical features of patients infected with 2019 novel coronavirus in Wuhan, China. Lancet 395, 497–506. 10.1016/S0140-6736(20)30183-5 31986264 PMC7159299

[B10] Jiang Y. Wang H. Chen Y. He J. Chen L. Liu Y. (2020). Clinical data on hospital environmental hygiene monitoring and medical staffs protection during the coronavirus disease 2019 outbreak. medRxiv [Preprint]. 10.1101/2020.02.25.20028043

[B11] Lamers M. M. Beumer J. van der Vaart J. Knoops K. Puschhof J. Breugem T. I.. (2020). SARS-CoV-2 productively infects human gut enterocytes. Science 2020:eabc1669. 10.1126/science.abc1669 32358202 PMC7199907

[B12] Li Z. Wu M. Guo J. Yao J. Liao X. Song S. (2020). Caution on kidney dysfunctions of 2019-nCoV patients. medRxiv 2020.02.08.20021212 10.1101/2020.02.08.20021212

[B13] Liu J. Li S. Liu J. Liang B. Wang X. Wang H.. (2020). Longitudinal characteristics of lymphocyte responses and cytokine profiles in the peripheral blood of SARS-CoV-2 infected patients. EBioMedicine 55:102763. 10.1016/j.ebiom.2020.102763 32361250 PMC7165294

[B14] Liu Y. Gayle A. A. Wilder-Smith A. Rocklöv J. (2020). The reproductive number of COVID-19 is higher compared to SARS coronavirus. J. Travel. Med. 27:taaa021. 10.1093/jtm/taaa021 32052846 PMC7074654

[B15] Lu C. W. Liu X. F. Jia Z. F. (2020). 2019-nCoV transmission through the ocular surface must not be ignored. Lancet 395:e39. 10.1016/S0140-6736(20)30313-5 32035510 PMC7133551

[B16] Lu R. Zhao X. Li J. Niu P. Yang B. Wu H.. (2020). Genomic characterisation and epidemiology of 2019 novel coronavirus: implications for virus origins and receptor binding. Lancet 395, 565–574. 10.1016/S0140-6736(20)30251-8 32007145 PMC7159086

[B17] Mahallawi W. H. Khabour O. F. Zhang Q. Makhdoum H. M. Suliman B. A. (2018). MERS-CoV infection in humans is associated with a pro-inflammatory Th1 and Th17 cytokine profile. Cytokine 104, 8–13. 10.1016/j.cyto.2018.01.025 29414327 PMC7129230

[B18] Pan Y. Zhang D. Yang P. Poon L. L. M. Wang Q. (2020). Viral load of SARS-CoV-2 in clinical samples. Lancet Infect. Dis. 20, 411–412. 10.1016/S1473-3099(20)30113-4 32105638 PMC7128099

[B19] Rothe C. Schunk M. Sothmann P. Bretzel G. Froeschl G. Wallrauch C.. (2020). Transmission of 2019-nCoV Infection from an asymptomatic contact in Germany. N. Engl. J. Med. 382, 970–971. 10.1056/NEJMc2001468 32003551 PMC7120970

[B20] Sun X. Zhang X. Chen X. Chen L. Deng C. Zou X. (2020). The infection evidence of SARS-COV-2 in ocular surface: a single-center cross-sectional study. medRxiv [Preprint]. 10.1101/2020.02.26.20027938 PMC7194535 32289466

[B21] Wan Y. Shang J. Graham R. Baric R. S. Li F. (2020). Receptor recognition by novel coronavirus from Wuhan: an analysis based on decade-long structural studies of SARS. J. Virol. 94, e00127–e00120. 10.1128/JVI.00127-20 31996437 PMC7081895

[B22] Wang D. Hu B. Hu C. Zhu F. Liu X. Zhang J. (2020). Clinical characteristics of 138 hospitalized patients with 2019 novel coronavirus-infected pneumonia in Wuhan, China. JAMA 323, 1061–1069. 10.1001/jama.2020.1585 PMC7042881 32031570

[B23] Wang W. Tang J. Wei F. (2020). Updated understanding of the outbreak of 2019 novel coronavirus (2019-nCoV) in Wuhan, China. J. Med. Virol. 92, 441–447. 10.1002/jmv.25689 31994742 PMC7167192

[B24] WHO (2020). Coronavirus Disease (COVID-2019) Situation Reports-127. 1–17. 10.18772/26180197.2020.v2nSIaMR

[B25] Wong C. K. Lam C. W. Wu A. K. Ip W. K. Lee N. L. Chan I. H.. (2004). Plasma inflammatory cytokines and chemokines in severe acute respiratory syndrome. Clin. Exp. Immunol. 136, 95–103. 10.1111/j.1365-2249.2004.02415.x 15030519 PMC1808997

[B26] Wrapp D. Wang N. Corbett K. S. Goldsmith J. A. Hsieh C.-L. Abiona O.. (2020). Cryo-EM structure of the 2019-nCoV spike in the prefusion conformation. Science 367, 1260–1263. 10.1126/science.abb2507 32075877 PMC7164637

[B27] Wu A. Peng Y. Huang B. Ding X. Wang X. Niu P.. (2020). Genome composition and divergence of the novel coronavirus (2019-nCoV) originating in China. Cell Host Microbe. 27, 325–328. 10.1016/j.chom.2020.02.001 32035028 PMC7154514

[B28] Wu C. Hu X. Song J. Du C. Xu J. Yang D. (2020). Heart injury signs are associated with higher and earlier mortality in coronavirus disease 2019 (COVID-19). medRxiv [Preprint]. 10.1101/2020.02.26.20028589

[B29] Wu F. Zhao S. Yu B. Chen Y. M. Wang W. Song Z. G.. (2020). A new coronavirus associated with human respiratory disease in China. Nature 579, 265–269. 10.1038/s41586-020-2008-3 32015508 PMC7094943

[B30] Xia J. Tong J. Liu M. Shen Y. Guo D. (2020). Evaluation of coronavirus in tears and conjunctival secretions of patients with SARS-CoV-2 infection. J. Med. Virol. 92, 589–594. 10.1002/jmv.25725 32100876 PMC7228294

[B31] Yeo C. Kaushal S. Yeo D. (2020). Enteric involvement of coronaviruses: is faecal-oral transmission of SARS-CoV-2 possible? Lancet Gastroenterol. Hepatol. 5, 335–337. 10.1016/S2468-1253(20)30048-0 32087098 PMC7130008

[B32] Zhang H. Kang Z. J. Gong H. Y. Xu D. Wang J. Li Z. F. (2020). The digestive system is a potential route of 2019-nCov infection: a bioinformatics analysis based on single-cell transcriptomes. bioRxiv [Preprint]. 10.1101/2020.01.30.927806

[B33] Zheng M. Song L. (2020). Novel antibody epitopes dominate the antigenicity of spike glycoprotein in SARS-CoV-2 compared to SARS-CoV. Cell. Mol. Immunol. 17, 536–538. 10.1038/s41423-020-0385-z 32132669 PMC7091851

[B34] Zhou P. Yang X. L. Wang X. G. Hu B. Zhang L. Zhang W.. (2020). A pneumonia outbreak associated with a new coronavirus of probable bat origin. Nature 579, 270–273. 10.1038/s41586-020-2012-7 32015507 PMC7095418

[B35] Zhou Y. Zeng Y. Tong Y. Chen C. (2020). Ophthalmologic evidence against the interpersonal transmission of 2019 novel coronavirus through conjunctiva. medRxiv [Preprint]. 10.1101/2020.02.11.20021956

[B36] Zhu N. Zhang D. Wang W. Li X. Yang B. Song J.. (2020). A novel coronavirus from patients with pneumonia in China, 2019. N. Engl. J. Med. 382, 727–733. 10.1056/NEJMoa2001017 31978945 PMC7092803

